# Metabolic Reprogramming of Liver Fibrosis

**DOI:** 10.3390/cells10123604

**Published:** 2021-12-20

**Authors:** M. Eugenia Delgado, Beatriz I. Cárdenas, Núria Farran, Mercedes Fernandez

**Affiliations:** IDIBAPS Biomedical Research Institute, University of Barcelona, 08036 Barcelona, Spain; iralde@clinic.cat (B.I.C.); nuface188@gmail.com (N.F.)

**Keywords:** HSC, CPEB4, RBPs, HCC, fibrosis, myofibroblast, obesity, ECM, metabolism, inflammation

## Abstract

Liver fibrosis is an excessive and imbalanced deposition of fibrous extracellular matrix (ECM) that is associated with the hepatic wound-healing response. It is also the common mechanism that contributes to the impairment of the liver function that is observed in many chronic liver diseases (CLD). Despite the efforts, no effective therapy against fibrosis exists yet. Worryingly, due to the growing obesity pandemic, fibrosis incidence is on the rise. Here, we aim to summarize the main components and mechanisms involved in the progression of liver fibrosis, with special focus on the metabolic regulation of key effectors of fibrogenesis, hepatic stellate cells (HSCs), and their role in the disease progression. Hepatic cells that undergo metabolic reprogramming require a tightly controlled, fine-tuned cellular response, allowing them to meet their energetic demands without affecting cellular integrity. Here, we aim to discuss the role of ribonucleic acid (RNA)-binding proteins (RBPs), whose dynamic nature being context- and stimuli-dependent make them very suitable for the fibrotic situation. Thus, we will not only summarize the up-to-date literature on the metabolic regulation of HSCs in liver fibrosis, but also on the RBP-dependent post-transcriptional regulation of this metabolic switch that results in such important consequences for the progression of fibrosis and CLD.

## 1. Introduction

Chronic liver disease (CLD) is nowadays one of the biggest threats to public health with an incidence of more than 29 million people in the European region alone [1]. Indeed, hepatic liver cancer is the sixth most common cancer worldwide [2] and in 2017 alone, cirrhosis caused more than 1.32 million deaths worldwide [3]. Worryingly, this incidence is expected to increase more than 25% in the next 20 years [2]. Surgery and liver transplantation are currently the only chances of long-term survival of patients with severe liver diseases. However, the availability of an appropriate donor and the overall cost of the procedure limits its broad application [4]. Consequently, there is a huge need for the search of treatment alternatives, as well as to develop new strategies for the prevention and treatment of CLD and cancer.

While different aetiologies can lead to CLD, a manifestation of in all these conditions is the development of fibrosis. Patients with progressive liver fibrosis due to excessive liver damage might develop cirrhosis and finally succumb to liver failure [5]. Hepatic fibrosis also generates a permissive micro-environment for the development of tumorigenic nodules through mechanisms that are still under debate [6], but nevertheless, increasing the risk for cancer [7]. Liver fibrosis is then an essential component of CLD. Unfortunately, despite the international efforts and due to the complexity and heterogenicity of the pathology among patients and aetiologies (further explored in the following sections), there are no current highly effective therapies that directly target the attenuation or reversal of liver fibrosis [8]. In this regard, a better understanding of the fibrosis-associated molecular and cellular mechanisms might provide new aspects in the diagnosis and treatment of CLDs.

In this review, we aim to summarize the main components and mechanisms that are involved in the progression of liver fibrosis. We will provide special focus on the metabolic regulation of the key effectors of fibrogenesis, hepatic stellate cells (HSCs), and their role in the progression of fibrosis. Of note, since fibrosis is a process where intrinsic and microenvironment mechanisms control cell fate decisions, it is considered a highly dynamic process that requires a fast response to properly accommodate the required energetic demands within the cells involved. It is noteworthy how, in recent years, ribonucleic acid (RNA) binding proteins (RBPs) and other RNA-regulators have been shown to play an important role in this process [9]. Here, we will summarize the up-to-date literature regarding not only on the metabolic regulation of HSCs in liver fibrosis, but also on the post-transcriptional regulation of this metabolic reprogramming that can result in such important consequences for the progression of fibrosis and CLD. 

## 2. Fibrogenesis and Chronic Liver Diseases

**Figure 1:**
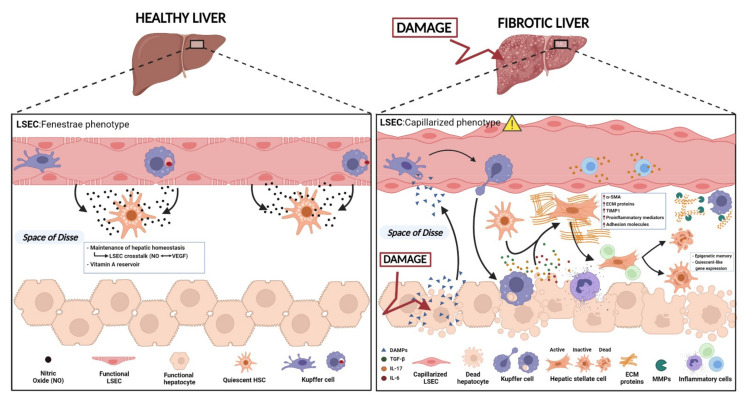
Progression of hepatic fibrosis. Under physiological conditions, HSCs are found in a quiescent state where they function as pericytes and reservoirs of retinol (vitamin A). Retinol, together with other lipids, is stored in perinuclear cytoplasmic lipid droplets. Liver sinusoidal endothelial cells (LSECs) retain HSCs quiescent state by the releasing of nitric oxide (NO). When the liver is injured, HSCs transdifferentiate into a myofibroblast-like cell with a high proliferative and secretory phenotype. This occurs as a consequence of different factors. Firstly, LSECs change their phenotype into a capillarized structure while stopping the release of NO. Damaged hepatocytes release damage-associated molecular patterns (DAMPs) that attract and activate Kupffer cells and other inflammatory cells. These macrophages go to the injury site and release pro-inflammatory and pro-fibrogenic cytokines such as TGF-B, IL-17, and IL-6, inducing the activation of HSCs. In this activated state, HSCs acquire a high proliferative, secretory phenotype, where the perinuclear lipid droplets are lost and high levels of alpha smooth muscle actin (α-SMA) are transcribed in an attempt to help the cell migrate to the site of injury. Together with ECM molecules, the activated HSCs secrete molecules of tissue inhibitor of metalloproteinase-1 (TIMP1) to control matrix degradation. Cytokines and growth factors that help to repair injured liver tissue, as well as different pro-inflammatory mediators and adhesion molecules are secreted to recruit resident and circulating immune cells, thus further contributing to the perpetuation of fibrosis. The figure was created with BioRender.com.

Liver fibrosis is an excessive and imbalanced deposition of fibrous extracellular matrix (ECM) that is mostly formed by crosslinked collagens (type I and type III), fibronectin, as well as elastin fibers, glycoproteins, and mucopolysaccharides, among others [10,11] (Figure 1). The main secretors of ECM proteins are activated myofibroblasts (MFBs) that, upon injury, replace normal liver tissue, remodeling the physiological architecture of this organ [12]. There are two general types of chronic liver injuries can lead to hepatic fibrosis: hepatotoxic injuries, due to chronic damage of hepatocytes (such as viral infection, alcohol intake, or metabolic syndromes) and cholestatic injuries that are caused by the obstruction of bile flow (as in biliary cholangitis and biliary atresia, among others) (reviewed in [5]). Not so long ago, the most common aetiologies leading to fibrosis were due to viral infections. These were the cases for the hepatitis B virus (HBV) and hepatitis C virus (HCV) infections. In 2017 alone, infection with these two virus were the major causes of liver fibrosis and contributed to around 50% of all cirrhosis and hepatocellular carcinoma (HCC) cases [3]. However, due to the growing obesity epidemic, non-alcoholic fatty liver disease (NAFLD) and non-alcoholic steatohepatitis (NASH) are considered the major precursors of fibrosis and liver failure nowadays [13,14]. The incidence of these obesity-derived diseases is so prevalent worldwide that experts have recently proposed a more specific term that is referred the metabolic-associated fatty liver disease (MAFLD). This new condition is defined as a cluster of fatty liver diseases that are associated with metabolic dysfunction [15] and it is considered a better criteria to identify patients with significant fibrosis via non-invasive methods [16]. Other less common diseases that can lead to excessive fibrosis and cirrhosis eventually, include autoimmune hepatitis, hemochromatosis, Wilson’s disease, and primary and secondary biliary cholangitis [17].

Fibrosis is thus a mechanism that is associated with tissue repair that, by isolating the wounded zone and supporting the spreading of growth factors and cytokines, enables the renewal of the damaged cells [18]. Thanks to the unique regenerative capacity of the liver, fibrosis is a reversible process. Once the source of injury is removed, fibrosis regression can occur. However, when fibrosis fails to terminate and resolve itself, it can lead to fatal consequences. The “point of no return” is considered when excessive ECM accumulation results in severe architectural distortion, leading to vascular collapse and portal hypertension. It is at this stage when significant regression is less likely to occur (reviewed in [19]). This is the case for CLD, with the accumulation of ECM persisting in time and leading to the generation of a fibrous scar which impairs the hepatic physiological functions. Cirrhosis, for instance, is a risk factor for several life-threatening complications such as portal hypertension, hepatic failure, and HCC, as reviewed in [20]. However, even clinical cirrhosis is considered a reversible process [21,22]. A meaningful regression of fibrosis might occur after the deletion of the trigger and removal of the underlying disease process [23]. In this line, the combination of improved therapy and vaccinations against HBV, together with the arrival of new antivirals against HCV infection, have considerably decreased the prevalence of liver fibrosis and cirrhosis due to these injuries, at least in the Western World [19,24]. In contrast, the lifestyle changes that are necessary for the improvement of the metabolic syndrome-associated NAFLD/NASH and obesity, are hard to implement. And unfortunately, the incidence of fibrotic livers due to obesity and metabolic conditions still persists around the globe [17].

The process of fibrosis is orchestrated mainly by MFBs, but implies the interaction of several factors from parenchymal and non-parenchymal cells [5] (Figure 1). Indeed, non-parenchymal cells (all cell types but hepatocytes) are the main cells deciding whether the fibrous scar is dissolved or progresses into an advanced stage [25]. In this regard, the interaction between MFBs with hepatocytes or the liver sinusoidal endothelial cells (LSECs) and other non-parenchymal cells, including Kupffer cells (KC) and lymphocytes, has been considered essential for the progression of fibrosis [25]. Nevertheless, MFBs have been described as the main cells that are responsible for ECM deposition in liver fibrosis and they consequently represent a primary target in antifibrotic therapies [26]. Hepatic resident MFBs are quiescent cells that become activated upon stimuli, including specific growth factors and cytokines that are released by the innate immune system. This myofibroblastic phenotype is characterized by high proliferative, synthetic, and contractile capacity (reviewed in [27] and is further explored in the following sections). In addition to the architectural disruption of vessels due to the ECM deposition, the contracted phenotype of MFBs leads to a rise in portal pressure [28,29,30], that, in turn, results in several complications, such as hydropic decompensation and bleeding events [31]. MFBs mostly consist of HSCs, together with a smaller population of portal fibroblasts and bone marrow-derived cells (fibrocytes and mesenchymal stem cells) (as reviewed in [32]). Upon injury, these cells activate and transdifferentiate into MFBs with a secretory phenotype. However, their exact contribution will depend on the underlying aetiology. Several studies using different labelling methods suggest that, in the case of hepatotoxic injury, HSCs represent more than 80% of the MFBs that are present in the damaged liver [33,34]. Otherwise, in the case of cholestatic injury, more than 70% of the MFBs were portal fibroblasts [34,35]. Thus, the exact source and contribution to the MFBs pool might highly depend on the evolution and type of injury [5,34].

ECM deposition is a dynamic process in terms of location, composition, and quantity of proteins and it presents a huge variability among tissues and diseases. Especially under pathological conditions, the ECM final protein profile and location highly depends on the underlying disease mechanism that triggered the fibrosis in the first place [19]. For instance, portal–portal and portal–central fibrosis have been related to viral infections, while metabolic-induced fibrosis is mainly produced around the centrilobular areas and usually forms a pericellular and perisinusoidal, “chicken-wire” pattern [36] (and is reviewed by [37]).

Of note, the assertion of fibrosis progression as linear along a continuum is an oversimplification. Actually, fibrosis progression tends to accelerate as the disease advances, especially toward more advanced stages [19,38]. This is also the case for the pathological ECM deposition during fibrosis. During the earlier stages of injury-induced fibrosis, the extracellular deposition of ECM is counterbalanced by proteolytic enzymes that promotes its own degradation, such as the matrix metalloproteinases (MMPs), which are released by platelet cells and other inflammatory and resident cells [11]. Following, at later stages of severe fibrosis progression, fibrolysis is overcome by fibrogenesis and it is characterized by the secretion of tissue inhibitors of MMPs (TIMPs) that are also released by HSCs and KC among others, that prevent the effect of the MMPs [39,40]. However, the MMPs functionality is not restricted to the degradation of ECM proteins. MMPs have also been described as an important regulator of the immune and inflammatory responses due to their effects in a variety of cytokines, chemokines, antimicrobial peptides, surface proteins, receptors, and junctional proteins, directly impacting the fibrogenic process [11,41]. Indeed, several therapeutic approaches have been developed using these ECM proteolytic enzymes as targets, highlighting their relevance in the fibrogenic process [11]. At later stages of fibrosis, the basement membrane also increases its mass and rigidity due to the gain in collagen deposition which varies depending on the type and degree of injury, while at the same time, gives rise to the characteristic periportal fibrosis [39,42]. Moreover, this collagen that is deposited in the extracellular space undergoes crosslinking by the lysyl-oxidase (LOX) enzyme primarily as it catalyses the formation of aldehydes from lysine residues in collagen and elastin, enhancing its reactivity and promoting further crosslinking [43,44]. Additionally, the sinusoidal endothelium transforms into a vascular endothelium by losing their *fenestrae*, thus significantly impairing the hepatic function [37,45] (Figure 1).

Earlier beliefs considered the ECM as a simple scaffold in the process of fibrogenesis. However, more recent advances showed how ECM holds both signalling and functional properties, suggesting a so far ignored relevance in the development of fibrosis. Indeed, ECM-specific conditions have been reported to be responsible for altering the behaviour and function of all resident liver cells [19,39]. In this regard, Olsen et al. [46] showed how primary rat HSCs became progressively myofibroblastic as substrate stiffness increased when cultured in a mechanically tunable polyacrylamide-based cell culture system. These results not only further support the role of ECM composition and the mechanical tension within the cell environment in the HSC differentiation [46,47], but they also provide an explanation for the described huge heterogeneity in fibrosis molecular patterns among patients and aetiologies [48].

Finally, and as previously described, when the liver injury is not maintained in time, fibrosis regression occurs. In this regard, macrophages and other resident and immune cells contribute to the degradation of the excessive ECM proteins by the secretion of MMPs, such as MMP1 (reviewed in [12]), as well as collagenases (as reviewed in [25]) (Figure 1). It is worth mentioning here that the reversibility of fibrosis does not necessarily imply that the liver can return to a completely healthy state. In this regard, the definition of fibrosis regression does not account for changes in the nodule size or the extent of terminal venular collapse. It also does not account whether altered types or distributions of collagen and other ECM components are back to the initial stage, or even if considerable changes in the liver architecture occur, especially after cirrhosis. It only refers to some degree of regression by the fibrotic fibers. At this point, still many questions remain. For instance, whether the regression of cirrhosis reduces the risk of developing HCC [19]. Further advances in the field are expected in the upcoming years. Of note, the liver regeneration capacity is lost progressively with time, suggesting this characteristic as a critical determinant for incipient liver failure [19]. Thus, a better understanding of how the ECM synthesis and degradation processes influence the biological outcomes in both health and disease, will rise more opportunities to develop effective drugs against CLD and fibrogenesis [11].

## 3. HSC-Dependent Molecular Mechanism of Liver Fibrosis

HSCs are a resident, non-parenchymal cell population that was originally identified by von Kupffer in 1876 [49]. They are the primary fibrogenic cell type in the injured liver, accounting for most of the MFBs that are involved in the process of hepatic fibrosis [50]. HSCs represent roughly the 10% of all hepatic cells and reside in the perisinusoidal space (space of Disse) as pericytes, between the hepatocytes and the LSECs, with extensions around the sinusoidal endothelium that, thanks to their endothelial *fenestrae,* allow the continuous exposure of HSCs to the blood flow [51,52]. In a healthy liver, chemical and mechanical stimulation maintain HSCs in a non-proliferative, quiescent phenotype [53]. HSCs in their quiescent state are also the main retinol (vitamin A) storing cells that, by accumulating retinyl esters (RE) within perinuclear lipid droplets, make the liver the reservoir of 60–95% of body’s vitamin A [9,54,55]. In normal conditions, retinol from the diet is esterified, transported, and hydrolyzed in hepatocytes [56,57]. After hydrolysis, the retinols are transferred to the HSCs for long-term storage. Once there, they undergo re-esterification by the enzyme lecithin retinol acyltransferase (LRAT) that transform RE for their storage in cytoplasmic lipid droplets that are present in quiescent and inactivated HSCs [58,59,60]. These lipid droplets accumulate around the nucleus and contain mainly triacylglycerols (TAG), RE, and LRAT [61,62]. During HSC activation, the lipid droplets change their content with less RE but more TAGs, they reduce in size, and change location from around the nucleus to the periphery of the cell [61,62]. These changes in TAGs are possible due to the modulated expression of diacylglycerol O-cyltransferase-1 (DGAT1) and adipose triglyceride lipase (ATGL), both are involved in the synthesis and breakdown of TAGs, as reviewed in [63]. Finally, during activation, stabilized RE is liberated from the lipid droplets by the action of retinyl ester hydrolases (REHs) [54] into the catabolism of fatty acids and β-oxidation. Therefore, activated HSCs do not present any cytoplasmic lipid droplet since they are used up as an energy source to proceed with the activation [9,61,64]. Interestingly, vitamin A treatment in HSCs has been shown to prevent culture-induced activation by partially inhibiting the expression of MFBs markers, α-SMA for instance, while maintaining quiescent markers [65]. This regulation is thought to be dependent on the activation of the nuclear receptors retinoic acid receptor-β (RARβ) and retinoid X receptor-α (RXRα) [66]. However, the exact mechanism between RE catabolism and the regulation of these transcription factors is still unknown [9]. Nevertheless, their role in non-fibrotic livers is not only limited to a simple vitamin A reservoir. A growing body of evidence acknowledges the role of HSCs as important regulators of hepatic growth, liver vasculature, immunity, and inflammation, as well as energy and nutrient homeostasis in both healthy and fibrotic livers (as reviewed in [67]).

**Figure 2:**
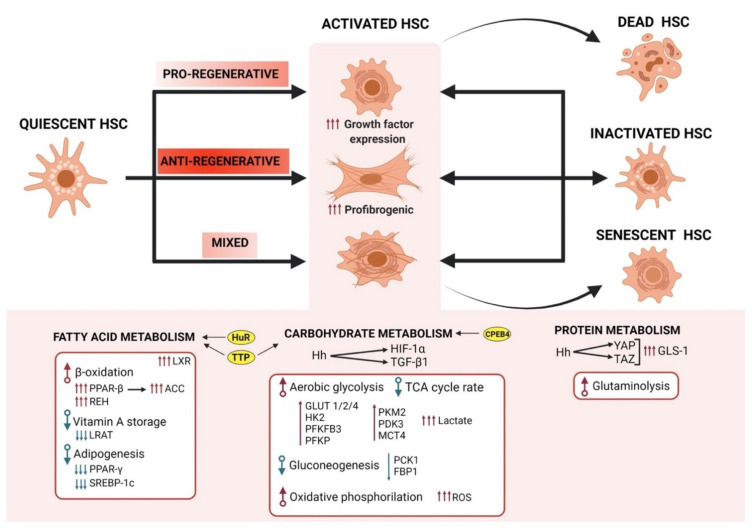
Metabolic reprogramming of HSCs during hepatic fibrosis. HSC activation is accompanied by a sequence of metabolic changes that allow the cell to meet their energetic demands that are required to materialize their newly acquired capabilities. Recent classification has subdivided activated cells depending on their expression profile, being pro-regenerative (increased growing factors), anti-regenerative (high pro-fibrogenic profile), and a subgroup with mixed phenotype. Once activated, genes that are related with retinol catabolism, such as retinyl ester hydrolase (REH), are upregulated, while enzymes that are involved in retinol esterification, such a lecithin retinol acyltransferase (LRAT), are downregulated. Consequently, lipid droplets disappear and they are metabolized to fuel the β-oxidation pathway. Enzymes that are involved in lipid metabolism such as the Liver X Receptors (LXRs) are upregulated and adipogenic regulators such as sterol regulatory element-binding protein 1 (SREBP-1c) are downregulated. Interestingly, the activated HSCs increase also their rate of aerobic glycolysis and the corresponding relevant enzymes while gluconeogenesis enzymes are reduced. Lactate accumulates intracellularly as well as the reactive oxygen species (ROS) and the oxidative phosphorylation pathway, while the tricarboxylic acid (TCA) pathway is downregulated. These metabolic changes are controlled, at least partially, by the activation of the Hedgehog (Hh) pathway via the expression of hypoxia-inducible factor 1-alpha (HIF-1α) together with transforming growth factor-β1 (TGF-β1). Glutaminolysis and protein metabolism is also upregulated alongside their rate-limiting enzymes, such as glutaminase-1 (GLS-1). This process is also regulated by the Hh pathway, this time via the transcription factor Yes-associated protein 1 (YAP) and its transcriptional cofactor TAZ. Interestingly, RNA-binding proteins (RBPs) such as polyadenylation-element-binding protein 4 (CPEB4), human antigen R (HuR), and tristetraprolin (TTP) have been described as key regulators of these metabolic rewiring in HSCs. Once the liver damage is relieved, the activated HSCs could become inactivated, dead, or senescent followed by their elimination via the immune system. The figure created with BioRender.com.

HSCs characterization studies with single-cell transcriptomic methods have raised awareness about their initially ignored heterogenous nature [68,69,70]. Accordingly, different HSCs subpopulations that are associated to the portal or central vein were identified in the healthy liver, suggesting functional zonation of HSCs during the liver injury [69]. HSCs have been traditionally described to present three different phenotypes: quiescent, activated, and inactivated. However, although this paradigm has resulted very useful, new advances in the field highlighted the heterogeneity in both the quiescent and in the activated HSC population, leaving this classification obsolete [70]. Moreover, this classification does not help to accommodate intermediate or hybrid states of HSCs with variable capacity for activation or with divergent contributions to regeneration, cancer, and immunomodulation, among others. Therefore, within the activated status of HSCs, further classification has been accounted for that results in three more subpopulations; the pro-regenerative (high growth factor expression), anti-regenerative (pro-fibrogenic), and mixed [9] (Figure 2).

Once the liver injury takes place, a series of paracrine and autocrine signalling loops can directly or indirectly induce the HSC activation [35]. In cases of hepatotoxic injuries where the damage initiates in the pericentral area, pericentral HSCs transdifferentiate into proliferative MFBs through an epithelial-to-mesenchymal transition (EMT) process. In this process, mature epithelial cells transition into fully differentiated mesenchymal cells (fibroblasts or myofibroblasts) [5,71]. Otherwise, the periportal HSCs population increases their proliferative capacity but they do not transdifferentiate into collagen-producing MFBs [9,69]. These results suggest that the patterns of HSC activation overlap with the areas of injury while at the same time it opens a possibility to develop new strategies to target only the pathogenic collagen-producing cell population, with the minimum perturbation of the liver function [69].

Interestingly, there are some cell types that prevent the HSC activation and differentiation into MFBs, such is the case of LSECs. In a healthy liver, LSECs help to maintain the quiescent phenotype of HSCs by the secretion of paracrine factors, for instance nitric oxide (NO) among others [5,72,73]. However, once fibrosis starts, LSECs change their phenotype (from a *fenestrae* structure into a capillarized structure), ceasing the secretion of NO and thus, releasing the HSCs from their quiescent state [73].

As the liver injury is relieved, the number of HSC-derived MFBs decline either by undergoing cell death or through a reversion to an inactivated phenotype. In this regard, some studies suggest that HSC do not revert into their original phenotype, but they present unique epigenetic signatures that allow these HSCs subtype to re-activate more readily upon re-injury [9,74] (Figure 2).

When hepatocytes die in the process of fibrosis, there is a release of damage-associated molecular patterns, DAMPs (such as adenosine triphosphate (ATP), uric acid, cholesterol crystals, deoxyribonucleic acid (DNA) fragments, and fatty acids [75]) that induce the recruitment of resident and infiltrated macrophages, neutrophils, and natural killer (NK) cells, among other inflammatory cells (reviewed in [5]). These cells are able to produce and secrete pro-inflammatory and pro-fibrogenic stimuli that help the differentiation of HSCs into an activated phenotype as reviewed in [76]. Therefore, under noxious stimuli, several cytokines are released to the media, which can be sensed by HSCs and induce their transdifferentiation. These stimuli include transforming growth factor beta (TGF-β) [77], platelet derived growth factor (PDGF) [78], tumour necrosis factor alpha (TNF-α) [79], leptin [80], and various interleukins (IL) (including IL-6, IL-17, and IL-1β) [81]. These fibrogenic mediators lead to changes in the storage of vitamin A as it transforms into an energy source for activation [82], as well as the upregulation of the expression of intracellular proteins such as vimentin, α-smooth muscle actin (α-SMA), and other intracellular microfilaments [5]. The same way, activated HSCs contribute to the ECM to form the fibrous scar by secreting large amounts of proteins such as collagen I, III, and IV, fibronectin, laminin, and proteoglycans, as well as tissue TIMP1 to control the matrix degradation [70]. Finally, the activated HSCs proceed to their own secretion of pro-inflammatory mediators (TGFβ, C-X-C motif chemokine Ligand 12/CXCL12, monocyte chemoattractant protein 1/MCP1, IL-10, and IL-8) and adhesion molecules (vascular cell adhesion molecule 1/VCAM-1) that help with the recruiting of more inflammatory cells, perpetuating the pro-fibrogenic phenotype, and further highlighting the close association between fibrosis and inflammation [5,12].

Once the aetiological source of the injury is resolved, it results in a decrease of the pro-inflammatory cytokines and, in turn, in a decline of the activated HSCs. There are at least three mechanisms contributing to the clearance of the activated HSCs: apoptosis, senescence, and reversion or inactivation, as reviewed in [5] (Figure 2). Particularly, in a fibrotic model of carbon tetrachloride (CCl4)-induced murine liver injury, 50% of HSCs underwent apoptosis after the removal of the aetiological agent, suggesting that the rest of the HSCs can stably persist in the liver once inactivated [74,83]. Apoptosis takes place as an imbalance between the antiapoptotic signals (from TGF-β and TIMP1) and pro-apoptotic signals, including death receptor ligation (reviewed in [30]) and the downregulation of antiapoptotic proteins such as the B-cell lymphoma 2 (BCL2) family members [5,84]. This apoptosis initiation is also mediated by the production of interferon gamma (IFN-γ) by NK cells that limits the population of activated HSCs [85]. There is also an HSCs activated population that, once the liver injury is resolved, become senescent instead of undergoing cell death. These cells present a secretory phenotype that allow them to mediate in the immune surveillance and inflammation, such as the ligand for NK cell receptors MCP1, facilitating the removal of quiescent HSCs. But they also help in the resolution of fibrosis as they diminish the secretion and expression of ECM proteins [5,83,86,87]. However, it is not clear yet why these active HSCs escape cell death and acquire a senescent quiescent-like phenotype, allowing them to be incorporated to the restored tissue [83]. Quiescent cells otherwise, are significantly different from inactivated or reverted HSCs [70]. Reverted or inactivated HSCs present a restored expression of their pro-fibrogenic protein profile (including changes in collagen-1, α-SMA, TGF-beta receptor type-1 (TGFRI), and TIMP1 expression), while they do not express quiescent makers (such as perilipin 2 and adiponectin receptor-1). These differences are mostly due to the retained epigenetic memory that promotes their more effective conversion to a pro-fibrogenic phenotype [5]. Nevertheless, although quiescent and inactivated HSCs do not express the same protein pattern, their pro-inflammatory and pro-fibrogenic gene expression are significantly downregulated and they both completely revert their function as pericytes, including their role as storage of vitamin A in lipid droplets, as well as mediators of the liver vasculature [74].

Altogether, it is widely accepted that HSCs present highly relevant roles in all the stages of liver fibrosis. Thus, further clarification of the HSCs’ contribution to fibrosis could cause a rise in important knowledge with relevant consequences in the clinical approach against fibrogenesis and CLD [27].

## 4. Metabolic Reprogramming of HSC in Fibrogenesis

HSCs highly rely on a tight regulation of their energy expenditure that allow them to hold the pleiotropic roles of HSCs in fibrogenesis, while at the same time, successfully managing the intrinsic and microenvironmental mechanisms that condition their cell fate. Indeed, several authors highlight the requirements for HSCs to undergo metabolic reprogramming to meet the energy demands that are needed to transdifferentiate and to perform their newly acquired abilities (reviewed in [9]) (Figure 2). HSC activation, for instance, resembles the energy requirements that are observed in cancer cells, although the latter is more due to unregulated growth that is driven by genomic mutations than by a tightly controlled mechanism of differentiation [88]. Thus, the better understanding of HSCs metabolic regulation is evolving as a new priority in the field of liver fibrosis, with important consequences for pericyte metabolism in other tissues [9].

To obtain energy during their transdifferentiation to MFBs, HSCs have been described to accommodate important changes in carbohydrate catabolism, including upregulation of glycolysis [9] (Figure 2). Indeed, activated HSCs present an overall enhanced glycolytic flux compared to quiescent cells [60]. Glycolysis is the conversion of glucose to pyruvate, following to its transformation to either lactic acid (anaerobic glycolysis) or to acetyl-CoA and utilized in the tricarboxylic acid cycle (TCA) (oxidative phosphorylation). In the presence of oxygen, non-proliferating tissues metabolize glucose through oxidative phosphorylation, and only when oxygen is limited is glucose converted to lactate. However, cancer cells, as well as normal proliferative tissues, tend to convert most glucose to lactate, avoiding oxidative phosphorylation (aerobic glycolysis) and despite the oxygen availability or mitochondria functionality [89]. This effect was first observed by Otto Warburg when studying cancer cells and it is usually named after him since then [90]. Of note, aerobic glycolysis is less efficient than oxidative phosphorylation in generating ATP, which suggests that glycolytic intermediates might be more relevant for these cells that are undergoing aerobic glycolysis than the energy molecule itself [63,89]. Interestingly, activated HSCs seem to shunt their glycolysis pathway towards the production and accumulation of lactate. Chen et al. [91] showed that activated HSCs accumulate elevated intracellular levels of lactate, even in the presence of higher lactate export pump monocarboxylate transporter 4 (MCT4) expression. Importantly, inhibiting this intracellular lactate accumulation resulted in the conversion of MFBs to quiescent HSCs [91]. Therefore, these data place lactate as one of the most relevant factors in the activation and perpetuation of the MFBs phenotype and it suggests that HSC activation presents far more metabolic requirements than the generation of ATP [9]. Nevertheless, although their dependency on aerobic glycolysis for the transdifferentiation into the MFBs phenotype seems to be high, activated HSCs still require major energetic contributions from oxidative phosphorylation, as reflected by their increased number and activity of mitochondria [60,91]. This effect might not be only attributable on their dependency on the ATP that is generated due to the oxidative phosphorylation, but also to the enhanced reactive oxygen species (ROS) that are derived from the increased mitochondrial activity [9,60] (Figure 2). In this regard, ROS signalling is part of a feed-forward loop with TGF-β. While redox imbalances activate TGF-β and the fibrotic cascade, TGF-β in turns, induces redox imbalances that further contribute to the ROS generation [92,93]. Additionally, excessive ROS production can stimulate inflammatory cells, contributing to the development of fibrosis. At the same time, however, it can lead to the hepatocyte death and liver damage, thus also supporting the progression of fibrosis [60,91,94]. Regardless of this, a more specific role for mitochondrial ROS in HSC activation and biology has not been established yet [9].

As already mentioned, activated HSCs seems to rely more on glucose metabolism than their quiescent counterparts. Supporting this, primary culture-activated or immortalized rat HSCs present higher levels of glucose transporter proteins, including GLUT1 [91], GLUT2 [95], and GLUT4 [96]. In this line, data from immortalized human activated HSCs as well as primary murine HSCs after activation, presented an increase in proteins that are related to the intracellular processing of glucose, such as hexokinase 2 (HK2), fructose- 2,6-bisphosphatase-3 (PFKFB3), and pyruvate kinase (PK). These effects suggest that HSCs need to upregulate their glycolytic pathway to get fully active [97]. Together with this upregulation, activated HSCs present a downregulation of proteins that are involved in gluconeogenesis, including phosphoenolpyruvate carboxykinase-1 (PCK1) and fructose bisphosphatase-1 (FBP1) [91], as well as the shunting of central carbon metabolic products away from the TCA cycle in favour to the lactate accumulation. This is the case for the increased pyruvate dehydrogenase kinase 3 (PDK3) that is observed in activated HSCs [91] or the upregulation of pyruvate kinase M2 (PKM2), which promotes the shift towards aerobic glycolysis in HSC activation [98]. However, while these data come from experiments with primary or immortalized HSCs where activation occurs under cell culture conditions (and higher extracellular levels of glucose), the relevance of their conclusions still needs to be confirmed in physiological scenarios.

Of note, the observed increase in enzymes that are involved in aerobic glycolysis during HSCs transdifferentiation seems to be managed by the activation of the Hedgehog (Hh) pathway [91]. Hh is a key regulator in development that becomes activated again in adults during tissue repair processes. The Hh pathway presents a low rate in quiescent HSCs, orchestrates the HSCs metabolic reprogramming by, among other things, inducing the expression of hypoxia-inducible factor 1-alpha (HIF1α), a master modulator of glycolysis, thereby controlling the fate of HSCs [91]. However, HIF1α regulation has been described by other means in the liver physiology. For instance, increased HIF1α expression around the central vein has been associated with low liver oxygen tension (as reviewed by [99]). Thus, additional Hh-dependent targets might exist that present a relevant role in the regulation of aerobic glycolysis during HSCs activation.

During activation, HSCs suffer a heavy bioenergetic toll to fulfil all of their secretory functions, thus protein metabolism is also reprogrammed (Figure 2). Du et al. [100] revealed a preferential expression of genes that are involved in protein metabolism over carbohydrate metabolism, that is consistent with the concept of amino acids as the major energy source in proliferating populations. In this study, they showed that at least 38% of the genes that appeared differently expressed between quiescent and activated HSCs were related to protein metabolism. Moreover, they showed that these differences resulted in a metabolic shift towards enhanced glutaminolysis. Indeed, the inhibition of glutaminolysis disrupted transdifferentiation, underscoring the importance of glutaminolysis as an energy source for activated HSCs [9,100].

Glutaminolysis is the conversion of the amino acid glutamine into α-ketoglutarate, a TCA cycle intermediate that is typically observed in cancer cells and provides the ATP that is required for cell anabolism. This conversion is a two-step process, being glutaminase-1 (GLS-1) the first rate-limiting enzyme that is involved in this conversion. Indeed, HSCs that are undergoing transdifferentiation up-regulate GLS-1 and its expression colocalize with MFBs markers. Moreover, GLS-1 expression appears to increase in HSCs of liver samples of NASH and advanced fibrosis patients, suggesting that also in these cases, a higher dependency on the glutaminolysis exists to activate and to conserve their MFBs phenotype [101].

As seen previously in the dependency on HSCs on aerobic glycolysis, the pathways that seem to be mediating this increase in glutaminolysis is also the Hh pathway [100]. In this case, a downstream mediator of Hh, the Yes-associated protein 1 (YAP), that together with its transcriptional cofactor TAZ, regulate the GLS-1 expression in HSCs [100] (Figure 2). Of interest, contrary to the inhibition of glutamine, glucose deprivation does not result in the abrogation of the fibrogenic process, suggesting glutamine as a preferred energy source for the promotion and preservation of the MFBs phenotype [9]. Otherwise, it is unknown whether other anaplerotic pathways, such as those that are dependent on PCK1, which is known to be coordinated with GLS-1 upregulation, can also contribute to liver fibrosis in a paracrine manner, besides its role in the MFBs phenotype maintenance [102,103]. Still, these data highlight the necessity to include glutaminolysis and its intermediate metabolites as potential targets to consider for liver antifibrogenic therapies, such as those already suggested in fibrosis of other tissues [104].

As mentioned before, one of the hallmarks of HSC activation is the utilization of the vitamin A reservoirs to the catabolism of fatty acids for energy supply. To favor this mechanism, the expression of LRAT in activated HSCs is considerably diminished, which translates in a reduced vitamin A storage and the further progression of fibrosis [105]. Although the reduction mechanism of LRAT expression is not fully understood, it seems to be dependent on IL-1 expression. In this regard, KC-derived IL-1 was shown to potently downregulate the mRNA and protein levels of LRAT without affecting the protein stability, thereby favoring the mobilization of RE as well as the activation of primary HSCs [106]. Indeed, in a model of hepatotoxic liver fibrosis with thioacetamide injection, animals with a deletion of IL-1 showed significant protection with respect to their wild-type counterparts [106]. These results suggest IL-1 as an injury signal that is relevant for the initiation of HSC activation. However, the same authors do not discard the contribution of other factors, such as TNF-α, that are also highly relevant for the inflammatory response [106]. Further details would be required to fully elucidate how exactly IL-1 suppresses the transcription of LRAT at the molecular level.

The metabolism of lipid droplets during HSC activation provides fatty acids for β-oxidation. In this regard, the inhibition of mitochondrial fatty acid catabolism blocks HSC activation [107], highlighting the relevance of lipid metabolism in the biology of HSCs. Interestingly, HSC activation is also controlled by master transcriptional regulators of fatty acid content [9]. These regulators include the peroxisome proliferator-activated receptor gamma (PPARγ) [108] and sterol regulatory-element-binding protein-1 (SREBP-1c) [109] (Figure 2). Indeed, the ectopic expression of these two nuclear receptors can revert HSC activation, further supporting the role of fatty acid synthesis in the maintenance of the HSCs inactivated phenotype [110]. Moreover, the lipid-activated nuclear transcription factor liver X receptors (LXRs), as key regulators of cholesterol homeostasis and hepatic lipogenesis [111], have been shown to regulate HSC activation and thus, the susceptibility to fibrotic liver disease. However, the exact relative contribution of LXR signalling to HSC activation remains to be clarified [112]. During transdifferentiation and the induction of β-oxidation, PPAR-β gets activated and downregulates PPARγ and SREBP-1c and the overall free fatty acid content decline, consistent with the loss of an adipogenic phenotype [82]. PPAR-γ presents a role in keeping HSCs in a senescent phenotype and its downregulation has been shown to induce the activation of HSCs via the transcriptional regulation of different mediators of fibrogenesis (such as TNF-α and PDGF, among others) [82]. Another enzyme that is described to be relevant in the fatty acid metabolic reprogramming of HSCs that are undergoing transdifferentiation is the acetyl-CoA carboxylase (ACC) (Figure 2). ACC is a regulator of fatty acid β-oxidation and de novo lipogenesis (DNL) [61]. Some studies with primary HSCs showed how preventing DNL via inhibition of ACC resulted in a reduction of activation markers such as α-SMA and collagen production, while preventing glycolysis and oxidative phosphorylation [113]. Moreover, in a model of obesity/diethylnitrosamine-induced hepatotoxic injury, ACC inhibition ameliorated liver fibrosis, supporting the role of ACC as an important regulator of HSC activation [114]. Although the mechanism by which ACC and DNL regulate HSCs metabolism still needs to be clarified, these results show that inhibition of DNL promotes HSC quiescence and reduces hepatic fibrosis. Therefore, the HSCs lipid content and its metabolic control represent a key controller in the pathogenesis of liver fibrosis and CLD.

All in all, the metabolic reprogramming of HSCs physiology is a fundamental component in the regulation of fibrosis and a key mechanism in the control of CLD progression. The elucidation of the specific relationship between metabolism and fibrogenesis will give rise to new opportunities to fight the progression of this broad-established liver injury.

## 5. RBPs Regulation of HSC Metabolic Reprogramming

The understanding of all of the factors that are involved, as well as the mechanisms governing the metabolic interactions in HSCs and in fibrosis in general, have been a huge effort in the scientific liver community in their attempts to develop new therapies [35]. In this regard, while the transcriptional networks that regulate fibrogenesis and HSCs transdifferentiation are extensively studied ([9,115,116] among others), how RNA regulatory processes control the metabolic rewiring of fibrogenesis remains less understood [117].

A paramount of health is associated with a proper regulation and management of RNA, where disruption in these networks leads to the development of human diseases [118]. Disturbances in the dynamic control of RNA results in dysregulated protein expression and thus their associated biological functions. Unveiling these regulatory roles of RNA networks is important to clarify the pathogenesis of chronic diseases such as fibrosis, where the mechanisms are not fully understood. Indeed, recent publications highlight the role of RNAs as important regulators of stress responses and metabolic disruptions [119,120].

RNAs interact with proteins to form ribonucleoprotein (RNP) complexes, which comprise of tens of thousands of different RNA sequences and hundreds of different RBPs [121]. Conventional RBPs exert their function by binding to sequence and/or structural motifs in RNA via modular combinations of a limited set of structurally well-defined RNA-binding domains; however, others associate in a sequence-independent manner. Some authors consider these RBPs as “RNA clothes”, which ensure that different RNA regions (mainly 5′ and 3′ untranslated (UTR) and coding regions) become covered or exposed according to the requirements to progress through the different stages of the RNA life [121,122]. One of the main consequences of dysregulated RNA is the disruptions of the RNA-RBPs networks and the biological events that are associated, including RNA export and transport, RNA cleavage, maturation, and stability, as well as functional changes in RBPs itself [118]. Therefore, RBPs play a highly relevant and multifunctional role in every step of the RNA life cycle and their dysregulation has a strong impact in several diseases, including HCC development [123,124].

The nature of RBPs binding to RNA is dynamic and it changes constantly, with the composition of RNA interactomes context- and stimuli-dependent [121]. It is not surprising then that recent studies on RBPs and CLD highlight the dynamic nature of the RBP response and the profound impact that these interactions have in the overall disease progression. This is especially true in a background of metabolic disorders where a tight regulation is required to control the intrinsic and microenvironmental mechanisms that affect the cell fate decisions [117,119]. In this regard, many RBPs have been already described as essential, not only in the regulation of HSCs metabolic reprogramming, but also in the general mechanism of fibrosis [117,123,125,126].

AU-rich element-binding proteins (AUBPs) are a class of RBPs that, by binding to the 3′-UTR of mRNAs, can cause either their degradation, stabilization, or translational inhibition. AUBPs have been described as key actors in pathological processes such as NASH and fibrosis, since the expression of several AUBPs was strongly altered in patients that were suffering from these diseases (reviewed by [126]).

Among the AUBPs, the human antigen R (HuR) holds a prominent role in CLD and fibrosis (Figure 2). Also known as ELAV-like RNA binding protein 1 (ELAVL1), it is predominantly expressed in the nucleus, but translocates to the cytoplasm once activated [127]. HuR, has been shown to contribute to HSC activation and liver fibrogenesis in humans, as well as in a mouse model of CCl4-induced injury [128,129]. In this case, the HSC activation undergoes through the HuR-dependent regulation of Sphingosine kinase 1 (SphK1), an enzyme that catalyzes the generation of sphingosine 1-phosphate (S1P), a lipid mediator with both intracellular and extracellular action modes (reviewed in [130]). Indeed, the up-regulation of SphK1 plays a crucial role in CLD and, together with S1P, have been shown to regulate several relevant processes for the inflammation and the angiogenesis that are associated with fibrosis [131,132]. Interestingly, Ge et al. [133] demonstrate that TGF-β 1 promotes the association of HuR with SphK1 mRNA and prolongs the half-life of SphK1 mRNA by stimulating the cytoplasmic accumulation of HuR, at least in murine fibrotic (CCl4-induced injury) livers [133]. Another mechanism by which HuR was reported to regulate HSCs effects in fibrogenesis is through the alleviation of HSCs by promoting their death by ferroptosis [134]. This type of cell death occurs due to the excessive accumulation of ROS and β-oxidation-dependent redox imbalance [135]. In this regard, the upregulation of HuR resulted in the stabilization of the mRNA of beclin1, promoted autophagic-dependent ferritin degradation, and eventually led to the induction of ferroptosis [136]. Of note, although not summarized here due to space constrictions, the functions of autophagy are intimately connected to the regulation of metabolism, therefore it also presents a relevant role in the HSCs biology and regulation [137]. These data provide an explanation of how lipid metabolism regulates HSC activation, as well as it provides further evidence of the relevance of this mechanism in the overall liver fibrosis progression.

Another member from the AUBPs family with a relevant role in the HSCs activity is the RBP tristetraprolin (TTP, also known as zinc finger protein 36 homolog/ZFP36) [126] (Figure 2). The ubiquitous TTP is one of the best-studied RBPs that is involved in the regulation of the cytoplasmic mRNA fate [138]. TTP activation occurs via the lipid-related activation of LXR-dependent transcriptional regulation and has been reported to influence a wide variety of inflammatory processes [139,140]. TTP has been reported to regulate the mRNA of several cytokines, HIF1α, and MMP9 (reviewed in [126]). Therefore, it is not surprising that TTP function has been related to a set of hallmark characteristics of tumour progression and HCC [141]. In HSCs, TTP has been shown to elicit protective activity against ferroptosis-induced cell death by its binding to the 3′-UTR of the autophagy-related 16-like 1 (ATG16L1) mRNA, promoting its degradation and thus ferroptosis inhibition [126,142]. In immortalized human HSCs (LX2), TTP overexpression led to the destabilization of MMP2 and TNFα, promoting the LX2 death by apoptosis, while impairing the cell activation, proliferation, and migration that was induced by TGF-β exposure, still via an unclear mechanism [143].

RBPs are also described to regulate the metabolic reprogramming and glucose metabolism directly during HSC activation [97]. That is the case for PFKFB3, which mRNA is stabilized by polyadenylation-element-binding protein 4 (CPEB4) [97] (Figure 2). This RBP belongs to the cytoplasmic-polyadenylation element binding protein family that present the ability to both activate and repress mRNA translation. This family of proteins is composed of four paralogs (CPEB1–4) in vertebrates, where CPEBs2–4 are closely related and CPEB1 is the most distant member of the family [144]. CPEB4, for instance, is highly expressed in the liver and its essential role in the stress response during liver diseases has been recently reviewed [145]. Interestingly, recent studies from our group using a murine model of diet-induced obesity placed CPEB4 as an important regulator of adipose tissue expansion [146]. Thus, considering the sensitivity of HSCs to lipid biology, these data also suggest CPEB4 as an indirect contributor to the activated HSC phenotype. In fibrotic livers, however, CPEB4 maintains HSCs in a high glycolytic state, predisposing them to the activation. Findings demonstrate that CPEB4 polyadenylates PFKFB3 mRNA, activating its translation [97]. Although this might not be the only regulatory mechanism of PFKFB3 in HSCs, the results of this study suggest that the translational regulation either precedes or dominates over transcriptional control during liver fibrosis. Indeed, immortalized HSCs, where CPEB4 levels were downregulated via short-hairpin RNA (shRNA)-mediated silencing, failed to up-regulate PFKFB3, highlighting the role of this RBP in the HSCs metabolic reprogramming [97]. Furthermore, silencing CPEB4 in knockout mice in which liver disease has been induced prevents HSC activation and liver fibrosis. The sum of these findings puts the CPEB4-PFKFB3-dependent axis into the spotlight as a potential target for antifibrotic strategies. This is highly relevant since, considering the incidence of fibrosis worldwide, there is an urgent need for development of novel antifibrotic agents.

## 6. Conclusions and Future Directions

Collectively, the literature that was covered in this review outline the significance of HSCs biology in the progression and resolution of fibrosis. Of special relevance is the metabolic reprogramming that HSCs undergo to fulfil their pleiotropic hepatic functions in health and disease. However, metabolic reprogramming is not restricted to HSCs. There are other cells in the liver that undergo metabolic reprogramming to fine-tune their cellular responses, thereby meeting their energetic demands during fibrogenesis. This category includes, but is not restricted to, infiltrated macrophages and KC, T lymphocytes, as well as hepatocytes (reviewed in [116]).

In the case of macrophages and KC, restrictions in their glucose and glutamine availability have been reported to inhibit their secretory functions [147]. On the contrary, the alteration of lipid metabolism that expose KCs to abnormal levels of fatty acids results in an enhanced pro-inflammatory phenotype due to their accumulation of cytotoxic lipids [148]. This switch in polarization promotes an increase in the levels of pro-inflammatory mediators such as cytokines and chemokines, finally leading to a higher degree of liver fibrosis [149].

In the same line, changes in glucose metabolism through hepatocyte-specific loss of the gluconeogenic enzyme FBP1 results in hepatocyte secretion of the non-histone nuclear protein high-mobility group protein B1 (HMGB1) [150]. HMGB1 is a DNA-binding non-histone nuclear protein that has been reported to induce the activation of HSCs and plays a remarkable role in the recruitment of pro-inflammatory neutrophils to sites of the necrotic injury in the liver, further contributing also to the development of fibrosis [12,151]. Indeed, TGF-β-dependent pathways have also been shown to be key inducers of the shift to aerobic glycolysis in cancer cells [152], suggesting a link between this growth factor and the metabolic rewiring. Altogether, this evidence highlights how relevant the metabolic rewiring is in all the hepatic cells and how its regulation contributes to the physiology of this tissue. These data also highlight how the dysregulation of these processes can lead to fatal consequences for the organ such as liver fibrosis, cirrhosis, or even HCC. Interestingly, the better understanding of these regulatory processes affecting this metabolic switch have opened new opportunities for therapeutic intervention in the recent years [97,116]. Thus, thanks to the advances in the field, a brighter future is expected for patients with liver fibrosis and CLD.

In this review, we discussed the role of RBPs in the regulation of HSC activation, in some cases, providing an answer for protein interconnections that were, so far, based only in mere observational correlations. That is the case for the TTP role on the so far unclarified LXR-dependent regulation of several inflammatory process [112,140]. We also tried to highlight the role of RBPs in liver fibrosis and the metabolic regulation of chronic liver diseases where fine-tuning protein synthesis and the resulting pathological cellular phenotypes are of paramount importance. Here we summarize the main findings regarding the role of common RBPs in the regulation of the metabolic activation of HSCs. It is clear though, that despite the existence of limited knowledge, the topic is emerging as very relevant in the field with high hopes regarding the exploitation of the specific RNA-RBP interactions in the discovery of a novel class of drugs against CLD and metabolic disorders [119]. Nonetheless, the effect of RBPs is not limited to HSCs or even to their metabolic rewiring. Further studies suggest a relevant role in RBPs in the regulation of important mechanisms that also contribute to the progression of liver fibrosis. These mechanisms include cytokine and growth factor release [153,154], EMT transition [117], collagen regulation [155,156], or the immune regulation of HCC hepatocytes [157], among others. That is the case for TTP with several important effects in the different stages of fibrosis [119,126,135,158]. For instance, TTP has been shown to regulate the mRNA of several cytokines and chemokines (including IL-17, TNFα, IL-6, IL-1β, and CXCL1-2) and in several cell lines (macrophages, lymphocytes, endothelial cells, and MFB) [126,140,153]. Of note, our studies highlight the role of CPEB4 in the stress resolution of obesity-driven fibrosis and CLD [97,146,159,160,161]. Thus, it is tempting to speculate that future studies in RBPs and liver diseases will help us to elucidate the mechanism by which fibrosis initiates and perpetuates its pathology leading to CLD. Unresolved issues in this regard include the role of ECM deposition and its heterogeneity in the progression of the disease, or the pattern of the HSC activation along the injured areas, or even the exact metabolic requirements for the HSC activation and inactivation. The elucidation of these mechanisms could help to develop new targets and new strategies to deal with the fibrogenic pathology at early stages, thus preventing its devastating consequences. However, the exploration other unresolved mechanisms in the fibrogenic process could also help to boost the efficiency of current hepatic immunogenic therapies. That is the case, for instance, for the not fully understood process by which HSCs became senescent. The better understanding of this process could help to develop therapies to help immune cells to remove excessive HSC activation and, therefore, improve the overall prognosis of CLD. Indeed, some of these families of RPBs represent important signalling nodes with relevant implications in multifactorial and heterogenous diseases, such as NASH, fibrosis, and HCC. Thus, targeting these RNA-RBPs interactions could open a therapeutic window of opportunity to “kill two birds with one stone” [126]. In this regard, although originally suggested “undruggable” due to their lack of a binding pocket, high-throughput methods have allowed the identification of potential molecules that affect the RBPs-binding activity. Consequently, these current technical advances could help in the development of novel therapeutics targeting RNA-RBP interactions [119,126]. Extensive studies are now required to expand our knowledge in the field of hepatic RBPs and to confirm the great potential that RBPs targeting hold for future therapies of CLD and HCC [126].

## Data Availability

Not applicable.

## Abbreviations

Acetyl-CoA carboxylase: ACC; Adenosine triphosphate: ATP; Adenylate-uridylate (AU)-rich elements: AREs; Adipose triglyceride lipase: ATGL Arginase 1: ARG1; AU-rich element-binding proteins: AUBPs; Autophagy-related 16-like 1: ATG16L1; B-cell lymphoma 2: BCL2; C-C chemokine receptor type 2: CCR2 C-X-C Motif Chemokine Ligand 12: CXCL12; Carbon tetrachloride: CCl4; Chronic liver disease: CLD; Tricarboxylic acid cycle: TCA; Cytoplasmic polyadenylation-element-binding protein 4: CPEB4; Damage-associated molecular patterns: DAMPs; De novo lipogenesis: DNL; Deoxyribonucleic acid: DNA; Diacylglycerol O-cyltransferase 1: DGAT1; ELAV like RNA binding protein 1: ELAVL1; Endoplasmic reticulum: ER; Epithelial-to-mesenchymal transition: EMT; Extracellular matrix: ECM; Fructose bisphosphatase 1: FBP1; Fructose-2,6-bisphosphatase-3: PFKFB3; Glucose transporter 1: GLUT1; Glutaminase-1: GLS-1; Hedgehog pathway: Hh; Hepatic stellate cells: HSCs; Hepatitis B virus: HBV; Hepatitis C virus: HCV; Hepatocellular carcinoma: HCC; Hexokinase 2: HK2; High fat diet: HFD; High-mobility group protein B1: HMGB1; Human antigen R: HuR; Hypoxia-inducible factor 1-alpha: HIF-1α; Intercellular Adhesion Molecule 1: ICAM1; Interferon gamma: IFNγ; Interleukins: IL; Kupffer cells: KC; Lecithin retinol acyltransferase: LRAT; Liver sinusoidal endothelial cells: LSECs; Liver X receptor: LXR; Lysyl oxidase: LOX; Matrix metalloproteinases: MMPs; Metabolic-associated fatty liver disease: MAFLD; Monoacylglycerol lipase: MAGL; Monocarboxylate transporter 4: MCT4; Monocyte chemoattractant protein 1: MCP1; Myofibroblast: MFBs; Natural killer cells: NK cells; Nicotinamide adenine dinucleotide phosphate: NADPH; Nitric oxide: NO; NLR family pyrin domain containing 3: NLRP3; Non-alcoholic fatty liver disease: NAFLD; Non-alcoholic steatohepatitis: NASH; Phosphoenolpyruvate carboxykinase 1: PCK1; Phosphofructokinase platelet: PFKP; Platelet derived growth factor: PDGF; Proliferator-activated receptor: PPAR; Pyruvate dehydrogenase kinase 3: PDK3; Pyruvate kinase M2: PKM2; Pyruvate kinase: PK; Reactive oxygen species: ROS; Retinoic acid receptor-beta: RAR-β; Retinoid X receptor-alpha: RXR-α; Retinyl ester hydrolases: REHs; Retinyl esters: RE; Ribonucleic acid: RNA;Ribonucleoprotein: RNP; RNA binding proteins: RBPs; Short-hairpin RNA: shRNA; Sphingosine 1-phosphate: S1P; Sphingosine kinase 1: Sphk1; Sterol regulatory-element- binding protein-1: SREBP-1c; TGF-beta receptor type-1: TGFRI; Tissue inhibitors of metalloproteinases: TIMPs; TNF receptor 1: TNFR1; Transforming growth factor beta: TGF-β; Triacylglycerols: TAG; Tricarboxylic acid: TCA; Tristetraprolin: TTP; Tumour necrosis factor alpha: TNF-α;Unstraslated region: UTR; Vascular cell adhesion molecule 1: VCAM-1; Yes-associated protein 1: YAP; Zinc finger protein 36 homolog: ZFP36; α-Smooth muscle actin: α-SMA.
